# Effect of Killed PRRSV Vaccine on Gut Microbiota Diversity in Pigs

**DOI:** 10.3390/v14051081

**Published:** 2022-05-18

**Authors:** Fangfeng Yuan, Jaishree Sharma, Som G. Nanjappa, Christopher A. Gaulke, Ying Fang

**Affiliations:** 1 Department of Pathobiology, College of Veterinary Medicine, University of Illinois at Urbana Champaign, Urbana, IL 61802, USA; fy8@illinois.edu (F.Y.); jsharma5@illinois.edu (J.S.); nanjappa@illinois.edu (S.G.N.); 2 Carl R. Woese Institute for Genomic Biology, University of Illinois at Urbana Champaign, Urbana, IL 61802, USA

**Keywords:** porcine reproductive and respiratory syndrome virus, PRRSV killed vaccine, gut microbiota

## Abstract

Porcine reproductive and respiratory syndrome virus (PRRSV) is one of the most economically important pathogens affecting the global swine industry. Vaccination is still a main strategy for PRRSV control; however, host factors associated with vaccine efficacy remain poorly understood. Growing evidence suggests that mucosa-associated microbiomes may play a role in the responses to vaccination. In this study, we investigated the effects of a killed virus vaccine on the gut microbiome diversity in pigs. Fecal microbial communities were longitudinally assessed in three groups of pigs (vaccinated/challenged with PRRSV, unvaccinated/challenged with PRRSV, and unvaccinated/unchallenged) before and after vaccination and after viral challenge. We observed significant interaction effects between viral challenge and vaccination on both taxonomic richness and community diversity of the gut microbiota. While some specific taxonomic alterations appear to be enhanced in vaccinated/challenged pigs, others appeared to be more consistent with the levels in control animals (unvaccinated/unchallenged), indicating that vaccination incompletely protects against viral impacts on the microbiome. The abundances of several microbial taxa were further determined to be correlated with the level of viral load and the amount of PRRSV reactive CD4^+^ and CD8^+^ T-cells. This study highlights the potential roles of gut microbiota in the response of pigs to vaccination, which may pave the road for the development of novel strategies to enhance vaccine efficacy.

## 1. Introduction

The gut microbiota of mammalian animals contains trillions of diverse microorganisms, including bacteria, viruses, fungi, protozoa, and archaea [1]. Microbiome refers to the microbiota, their genomes, and the abiotic factors of a given habitat while microbiota refers to the specific microbes, although these two terms are often used interchangeably [2]. Beyond the wide range of essential and beneficial functions (breaking down nutrients, preventing pathogen colonization, etc.), gut microbiota has also been shown to have major impacts on metabolism, central nervous system function, inflammation disorders, etc. [3,4,5,6].

Despite the growing evidence of a link between microbiota and host immunity, its impact on immune responses to vaccination against infectious diseases is still poorly understood [7]. Microbiota is proposed to play key roles in the development and modulation of host immune systems [8]. Microbiota also contributes to the variation of vaccine efficacies in populations throughout the world and between individuals [7]. Interplay between gut microbiota and vaccination was also reported for vaccines against Rotavirus and Poliovirus [9,10]. Oral immunization of the rotavirus vaccine correlates with increased *Streptococcus bovis* and reduced species in the Bacterioidetes phylum of the fecal microbiota in infants [9]. A high relative abundance of Pseudomonadales was reported to be associated with low T cell responses and virus-specific IgG levels in serum after the oral immunization of infants with a poliovirus vaccine [10]. The potential mechanisms by which microbiota modulate immune responses to vaccination are to be determined but have been proposed to engage in multiple ways, including the natural adjuvant hypothesis (innate sensing of microbiota by pattern recognition receptors), immunomodulation by microbial metabolites (short-chain fatty acids or SCFAs), microbiota-mediated reprogramming of antigen-presenting cells, and microbiota-encoded cross-reactive antigens [11].

Porcine reproductive and respiratory syndrome (PRRS) is considered as one of the most costly diseases for the swine industry worldwide. The economic loss is about $600 million annually in the United States [12]. PRRS killed and modified live-attenuated vaccines are commercially available [13,14]. The modified live virus (MLV) vaccines have been widely used but cannot fully protect pigs against heterologous PRRSV infections, and they have failed to block viral transmission between animals [14,15]. The potential risk of virulence reversion is also a concern [16]. Non-infectious PRRSV vaccines, including killed virus vaccines, are safer to use but the vaccine efficacy needs to be improved [13]. New strategies are needed to develop innovative non-infectious PRRSV vaccines. The interaction between PRRSV infection/vaccination and microbiota could provide novel insights into microbiota-targeted intervention strategies and vaccine development. A previous study demonstrated that several gut microbiome characteristics are associated with an improved outcome in pigs co-infected with PRRSV and Porcine circovirus 2 (PCV2) [17]. Further studies are still to be conducted to identify the microbial signatures that correlate with PRRS vaccine efficacy and immunogenicity.

In the current study, we evaluated the effectiveness of a killed PRRSV vaccine and explored the gut microbiome characteristics associated with improved outcomes and vaccine-induced immunity in a nursery pig model. This study provides the fundamental knowledge for potentially employing microbiota in the future development of inactivated PRRS vaccines.

## 2. Materials and Methods

### 2.1. Cells and Viruses

PRRSV-2 isolate VR2332 (GenBank accession no. EF536003.1), the parental virus of a currently wide-used PRRS MLV vaccine (Ingelvac PRRS^®^ MLV), was used for vaccine preparation and all other experiments. MARC-145 cells were used for virus propagation. They were maintained in minimum essential medium (MEM, Gibco/Thermo Fisher Scientific, Waltham, MA, USA) supplemented with 8% fetal bovine serum (Sigma, Burlington, MA, USA) and antibiotics (100 µg/mL streptomycin, 100 U/mL penicillin) at 37 °C with 5% CO_2_.

### 2.2. Inactivated Vaccine Preparation

The killed PRRSV vaccine was prepared by inactivating the VR2332 virus using the binary ethyleneimine (BEI) method. Initially, virus stock was prepared by harvesting the cell culture supernatant from virus-infected cells, concentrated with the Macrosep Advance Centrifugal Devices (Pall, Westborough, MA, USA), and then diluted to a titer of 10^8^ TCID_50_/mL in MEM. A 0.1 M BEI stock was prepared by dissolving 2-bromoethylamine (Sigma, Burlington, MA, USA) in 0.175 M NaOH (Thermo Fisher Scientific, Waltham, MA, USA) for 1 h at 37 °C and stored at 4 °C. Virus inactivation was performed by incubating 10 mL of the virus with 1 mM BEI at 37 °C for 24 h with gently stirring. The remaining BEI was neutralized by incubation with 0.1 mM sodium thiosulphate (Sigma, Burlington, MA, USA) for 2 h at 37 °C. The complete loss of infectivity was confirmed by inoculating MARC-145 cells with the killed virus.

### 2.3. Animal Experiment

A total of 24 specific-pathogen-free (SPF) 4-week-old piglets were obtained from a certified PRRSV-negative herd from the Swine Research Center at the University of Illinois at Urbana-Champaign (UIUC). All pigs were randomly divided into three groups (n = 8) and housed separately in the Edward R. Madigan Laboratory (ERML) large animal facility at UIUC. Pigs were fed a regular diet that contains soybean, corn, whey and other growth additives formulated by the Animal Science laboratory at UIUC. Group 1 pigs were intramuscularly immunized with the prepared killed vaccine at a dose of 1 × 10^8^ TCID_50_ per pig, while group 2 and 3 pigs were mock-immunized with cell culture medium. At 14 days post-vaccination (14 dpv), all three groups were boosted using the same method as that in the primary immunization. Three weeks (35 dpv) after the boost, group 1 and 2 pigs were challenged with the live VR2332 virus at 5 × 10^4^ TCID_50_ per pig, while group 3 pigs were mock-challenged with PBS. All pigs were terminated at 10 days post-challenge (10 dpc; equivalent to 45 dpv). The pigs were observed daily, and clinical signs were evaluated by veterinary technicians in the animal facility, including the animal activity, feed consumption, body condition, and respiratory signs (coughing, sneezing, elaborated breathing, nasal discharge). Rectal temperature was measured daily after challenge. Fecal swabs were collected from each piglet at 0, 35, and 45 dpv. Serum samples were collected at 0, 3, 7, and 10 dpc, while whole blood samples were collected at 10 dpc. Fecal and serum samples were placed in an ice-cold container immediately after collection and stored in a −80 °C freezer after being shipped back to the lab. Whole blood samples were collected and stored at room temperature for preparation of flow cytometry analysis immediately after being shipped back to the lab. During necropsy, the lungs were evaluated for gross lesions using a method described previously [18]. The body weight of each piglet was recorded during the first day (0 dpv) and last day (45 dpv) of the study. The pig experiment was conducted according to the protocol approved by the Institutional Animal Care and Use Committee (IACUC) of the University of Illinois at Urbana-Champaign.

### 2.4. Quantification of Viral Load

For measuring viral load, serum samples from 3, 7, and 10 dpc were subjected to viral RNA isolation using a MagMAX™ Viral RNA Isolation Kit (Applied Biosystems™, Waltham, MA, USA) and qRT-PCR was subsequently performed using a real-time quantitative RT-PCR kit (Tetracore, Rockville, MD, USA) in the ABI 7500 real-time PCR system. A standard curve was established by using a ten-fold serial dilution of the positive control included in the Tetracore kit. The amount of viral RNA (copy numbers per mL) was calculated based on the standard curve.

### 2.5. Serum Neutralization Assay

The serum neutralization assay was performed as described previously [19]. Briefly, the terminal serum samples collected from all three groups at 10 dpc were heat-inactivated at 56 °C for 30 min. A two-fold serial dilution of serum samples was prepared and added to a 96-well plate (100 µL/well). An equal volume of VR2332 (200 TCID_50_) was added to each well of serum dilutions and mixed well. The mixture was incubated at 37 °C for 1 h. After incubation, the mixture was transferred to a 96-well plate containing 100% confluent MARC-145 cells. At 18 h post-infection (hpi), cells were fixed with 80% acetone and stained with PRRSV-specific monoclonal antibody SDOW17 (anti-nucleocapsid protein) [20]. Alexa Fluor 488 AffiniPure goat anti-mouse IgG (Jackson Immuno Research, West Grove, PA, USA) was used as the secondary antibody. Fluorescent foci of infected cells were counted using a phase-contrast fluorescent microscope and the neutralizing antibody titer was interpreted as the highest serum dilution at which more than 90% of virus infection was inhibited.

### 2.6. Flow Cytometry

To measure the frequencies of the PRRSV-specific T cell populations, peripheral blood mononuclear cells (PBMCs) were isolated from the pig blood using SepMate™-50 (Stemcell) and Lymphoprep™ Density Gradient Medium (Stemcell Technologies, Cambridge, MA, USA) following the manufacturer’s instructions. PBMCs (2 × 10^6^/well) were seeded on a 6-well plate. At 24 h post-seeding, cells were stimulated with VR2332 (2 × 10^6^ TCID_50_/well), and BD GolgiStop (4 µL per well) was added at 18 h post-stimulation. Positive control PBMCs were stimulated with PMA (20 ng/mL) and Ionomycin (1 µg/mL). At 24 h post-stimulation, surface and intracellular staining were performed. PBMCs were first surface labeled with PerCP-Cy™5.5 Mouse Anti-Pig CD3ε, BD Horizon™ BV421 Rat Anti-Mouse CD44, PE-Cy™7 Mouse Anti-Pig CD4a, and FITC Mouse Anti-Pig CD8a. After staining the surface markers, cells were fixed and permeabilized using a Cytofix/Cytoperm™ Fixation/Permeabilization Solution Kit (BD Biosciences, San Diego, CA, USA). Cells were washed and stained with BD Pharmingen^TM^ Mouse Anti-Pig IFNγ or PE-labeled mouse IgG1 as isotype control. Finally, the cells were washed, suspended in a 2% paraformaldehyde solution, and analyzed using a full-spectrum Cytek Aurora flow cytometer. Data were analyzed using the FlowJo v10 software (BD Biosciences, San Diego, CA, USA). The frequencies of cell population were calculated as a percentage of the live lymphocytes of PBMCs.

### 2.7. Genomic DNA Extraction, 16S Amplicon Library Preparation, and Metagenomic Sequencing

Fecal swabs collected at 0 dpv, 35 dpv (0 dpc), and 45 dpv (10 dpc) from each piglet were subject to microbial DNA extraction using the DNeasy PowerSoil Pro kit (QIAGEN, Germantown, MD, USA) according to the manufacturer’s instructions. An extra step of incubation at 65 °C for 10 min was added prior to bead beating to facilitate cellular lysis. The samples were subjected to bead beating for 20 m using a Vortex-Genie 2 and Vortex Adapter (QIAGEN, Germantown, MD, USA). The amplification of the 16S rRNA V4 region was performed with universal 16S rRNA primers targeting the V4 region [21]. The master mix composition was 13 µL PCR grade water, 10 µL DreamTaq (Thermo Fisher Scientific, Waltham, MA, USA), 0.5 µL of each sequencing primer, and 1 µL DNA. The PCR conditions were 95 °C for 3 m; 35 cycles of 95 °C for 45 s, 50 °C for 60 s, and 72 °C for 90 s; and 72 °C for 10 m followed by a 4 °C hold. Amplicons were visualized using gel electrophoresis and further purified using a QIAquick PCR Purification Kit (QIAGEN, Germantown, MD, USA). The concentration of amplicons in each sample was measured using the Qubit^®^ HS kit (Thermo Fisher Scientific, Waltham, MA, USA) and 200 ng of each sample was pooled. The pooled library was cleaned again and diluted for measuring the concentration. The final cleaned pool product was diluted to a concentration of 10 nM and submitted to the Roy J. Carver Biotechnology Center of UIUC for cluster generation and 250 bp paired-end sequencing using an Illumina MiSeq instrument. Both forward and reverse reads were input into DADA2 (v 1.20) for quality filtering, sequence variant calling, chimera filtering, and taxonomy assignment using the SILVA (v138.1) reference database [22,23]. All sequence data were deposited in the sequence read archive under the project accession numberPRJNA820854.

### 2.8. Microbial Community Analysis

Alpha and beta diversity were calculated using R, and the vegan package (v2.5.7). Principal component analysis (PCA, USA) was performed using prcomp (R, stats, v 4.1.1) and visualized using ggplot (R, ggplot2, v 3.3.5). Generalized linear mixed models (GLMM) quantified associations between the date of the sample collection, viral challenge, vaccination and alpha diversity (richness and Shannon entropy) while accounting for the random effect of sample ID (R, glmmTMB, v 1.1.2.3). Associations between the microbiome beta diversity (Bray-Curtis) and the date of the sample collection, viral challenge, and vaccination status were assessed using Permutational Multivariate Analysis of Variance (Adonis, R, vegan). Prior to analysis, sequence read counts were normalized with rarefaction to a depth of 5000 reads. Samples not meeting this read threshold were removed from subsequent analysis.

To identify the taxa whose abundance was significantly altered in response to vaccination or viral challenge, negative binomial general linear mixed models (glmmTMB) were used. Briefly, for each taxa, a null model containing only the date and the random effect of sample ID was constructed. An alternative model was then constructed consisting of taxa as the response with the date and the interaction between viral challenge and vaccination as predictors. An ANOVA was used to determine if the alternative model explained significantly more variation than the null. This enabled us to compensate for temporal variation in the data. Spearman correlations quantified associations between immune parameters and microbial abundances. The false discovery rate was controlled using Storey’s q-value (R, qvalue, v2.24.0), and a threshold of 0.2 was used unless otherwise mentioned [24].

## 3. Results

### 3.1. Clinical Presentation and Performance of Vaccinated and Non-Vaccinated Pigs

**Figure 1:**
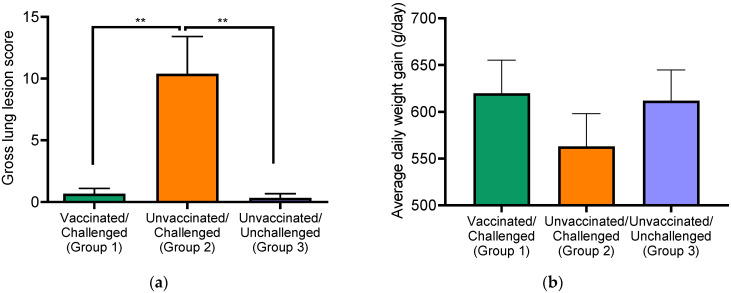
Comparison of gross lung lesion and body weight changes among different treatment groups of pigs. (**a**) Gross lung pathology. Gross lung lesion was evaluated using a published scoring system [18], in which each lobe of the lung was assessed for percentage of pneumonia and the scores of all lobes add up to represent the total lung pathology. (**b**) Average daily weight gain of pigs. Pigs from each group were weighed on the first and last day of the experiment. The average daily gain of body weight for each pig was calculated. Data are presented as means ± standard error. ** *p* < 0.001.

We assessed the efficacy of the killed PRRSV vaccine in a nursery pig model. Three groups of pigs were used, including group 1 pigs that were vaccinated and challenged with live PRRSV, group 2 pigs that were unvaccinated and challenged with live PRRSV, and group 3 pigs assigned as negative control (unvaccinated/unchallenged). No apparent clinical signs were observed in any of the experimental pigs. However, mild pathological lung lesions including interstitial pneumonia were observed in all group 2 pigs, while pigs in the vaccinated group (group 1) and negative control group (group 3) did not show much specific lung lesions (Figure 1a). Comparing the gross lung lesions among different groups of pigs, group 2 pigs had a significantly higher mean lesion score (10.40) than that of the other two groups (mean lesion score of 0.67 for group 1 pigs and 0.33 for group 3 pigs).

The vaccination appears to be able to maintain the performance of the pigs. At 10 dpc, vaccinated pigs (group 1) showed a body weight comparable to that of the negative control pigs (group 3). In contrast, group 2 pigs had a 49 g lower average daily weight gain than those of group 3 pigs (Figure 1b).

### 3.2. Viremia and Host Immune Responses

**Figure 2:**
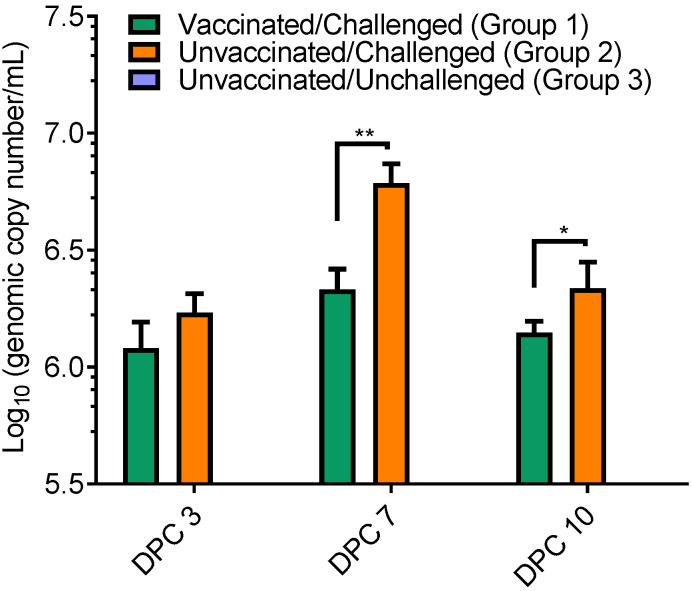
Comparison of viral loads in different groups of pigs throughout the time course of study. Real-time qRT-PCR was performed using serum samples collected at 3 dpc, 7 dpc, and 10 dpc. Viral RNA was quantified and interpreted as genomic copies per milliliter. Data are presented as means ± standard error. * *p* < 0.05, ** *p* < 0.001.

Viral RNA loads in serum samples were measured using real-time qRT-PCR. In comparison with the non-vaccinated group 2 pigs, vaccinated group 1 pigs showed consistently lower viral RNA loads throughout the 10 days post-challenge (dpc 3–10; Figure 2). Statistically, significant lower levels of viral RNA were obtained at 7 and 10 dpc in vaccinated pigs. As we expected, no viral RNA was detected in serum samples collected from the negative control group 3 pigs.

We further assessed the serum neutralizing (SN) antibody levels in serum samples collected at 10 dpc. In group 1 pigs, 6 out of 8 vaccinated pigs developed a detectable level of neutralizing antibody response (SN titer of 1:4–1:8). No SN antibody was detected in group 2 and group 3 pigs.

**Figure 3:**
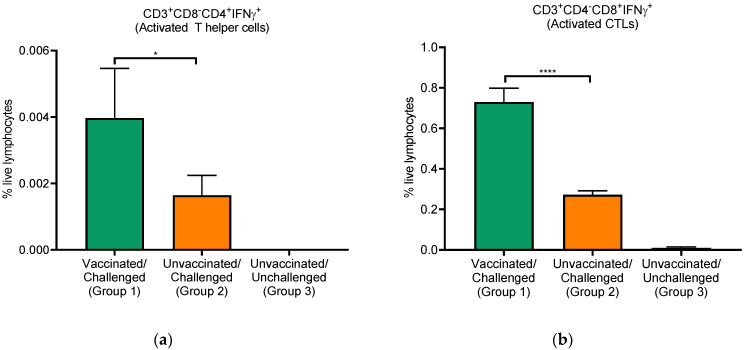
T cell responses in vaccinated and unvaccinated pigs. PBMCs collected from pigs at 10 dpc were isolated and stimulated with PRRSV-2 strain VR2332 at 1 moi. Cells were immunostained using antibodies against cell surface markers of CD3, CD44, CD4, and CD8, followed by intracellular IFNγ staining. IFNγ-secreting cells of CD3^+^CD8^−^CD4^+^ (Helper T cells; **a**) and CD3^+^CD4^−^CD8^+^ (Cytotoxic T cells; **b**) were calculated as a percentage of total live lymphocytes. Each bar is the mean value ± SEM. * *p* < 0.05, **** *p* < 0.0001.

Next, we compared the PRRSV-specific T cell responses in vaccinated and non-vaccinated pigs. PBMCs were isolated from blood samples collected on day 10 post-challenge. IFNγ-secreting lymphocyte subsets were detected by flow cytometry following ex vivo restimulation of PBMCs with the same virus (VR2332) used for vaccination/challenge. The subpopulations of T cells were identified based on a combination of cell surface markers, i.e., CD3^+^CD4^−^CD8^+^ (Cytotoxic T lymphocytes, CTLs) and CD3^+^CD8^−^CD4^+^ (Helper T cells). After staining surface markers, the cells were fixed and stained for intracellular cytokine and gated for respective IFNγ^+^ phenotypes. Percentages of cytokine^+^ T cells among live lymphocytes were assessed. As shown in Figure 3, the frequencies of IFNγ-secreting CD3^+^CD4^−^CD8^+^ and CD3^+^CD8^−^CD4^+^ cells were significantly higher in vaccinated pigs (group 1) than those from non-vaccinated (group 2) and negative control (group 3) pigs. These data suggested that the two major subsets of T cells were significantly higher in vaccinated pigs than in the other groups, suggesting vaccine-mediated virus-specific activation of adaptive T-cell immunity.

### 3.3. Fecal Microbial Community Composition

**Figure 4:**
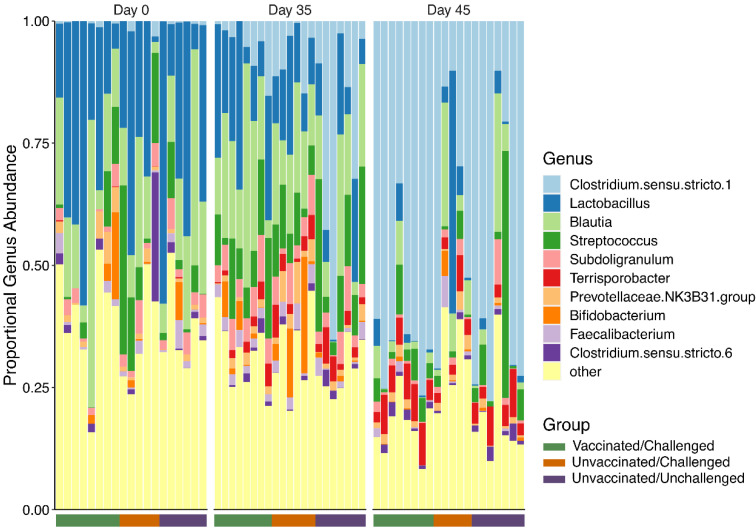
Relative microbial abundance of fecal microbiota in genus level. Genus level fecal microbial composition of all piglets at 0 dpv, 35 dpv, and 45 dpv was calculated. The legend on the right represents each individual microbial genus in different colors. The top two genus include *Clostridium* in light blue and *Lactobacillus* in dark blue.

To assess the composition of the fecal microbiome in all groups of pigs, fecal swab samples were subjected to 16s rRNA amplification and next-generation sequencing. A total of 2,407,574 high-quality chimera filtered reads were generated (mean/sample = 40,126 ± 8508) and represented 3352 amplicon sequence variants (ASV) and 171 genera. On day 0, prior to vaccination, the pig fecal microbiomes were dominated by three genera: *Lactobacillus*, *Blautia*, and *Streprococcus*. The relative abundance of *Clostridium sensu stricto 1* increased substantially in the majority of animals between 0 dpv (>5%) and 45 dpv (~75%) (Figure 4). This increase was accompanied by decreases in *Lactobacillus*, *Blautia*, and *Streprococcus*. These trends indicated that temporal variability contributed to community composition.

### 3.4. Fecal Microbiome Diversity

**Figure 5:**
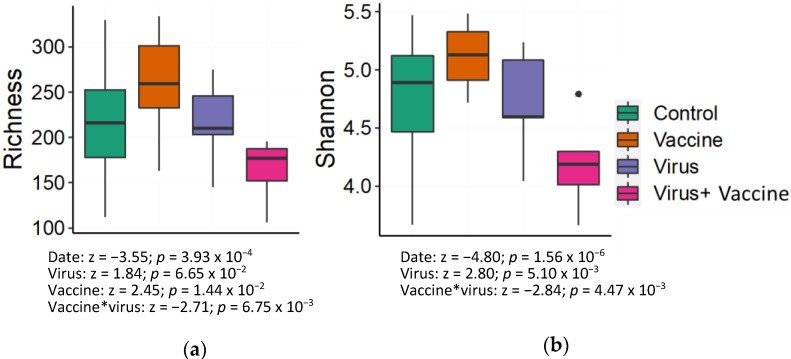
Alpha diversity of fecal microbiota. Boxplot depicting species richness (**a**) and Shannon diversity index (**b**) were graphed using all group pig samples from 0 dpv, 35 dpv, and 45 dpv (10 dpc). Legend on the right shows four factors including control, vaccine, virus, and virus + vaccine. Black dot in panel b indicates a statistical outlier (i.e., Quartile 3 + 1.5 × Interquartile range).

The alpha-diversity quantifies microbial community diversity within individual samples. Two common measures of alpha diversity are taxonomic richness which quantifies the total number of species and Shannon entropy which quantifies both the richness of a community and the evenness, with which the abundance is distributed across species. General linear mixed-effects models were used to quantify how time, vaccination, and viral infection impact alpha diversity. Consistent with the observed changes in genera composition (Figure 4), richness was negatively associated with time (z = −3.55, *p* = 3.93 × 10^−4^), indicating that fecal microbial richness decreases along with pig growth (Figure 5a). The independent effects of both the virus (z = 1.84; *p* = 6.65 × 10^−2^) and the vaccine (z = 2.45; *p* = 1.44 × 10^−2^) correlate with increased richness; however, these effects only reached significance for vaccination (*p* < 0.05). Interestingly, the interaction between the vaccination and viral challenge led to a significant decrease in microbial richness (z = −2.71; *p* = 6.75 × 10^−3^). Similar effects were observed in Shannon entropy (Figure 5b). Entropy decreased with time (z = −4.80; *p* = 1.56 × 10^−6^), while the virus infection increased entropy (z = 2.80; *p* = 5.10 × 10^−3^) and PRRSV challenge after vaccination decreased entropy (z = −2.84; *p* = 4.47 × 10^−3^).

**Figure 6:**
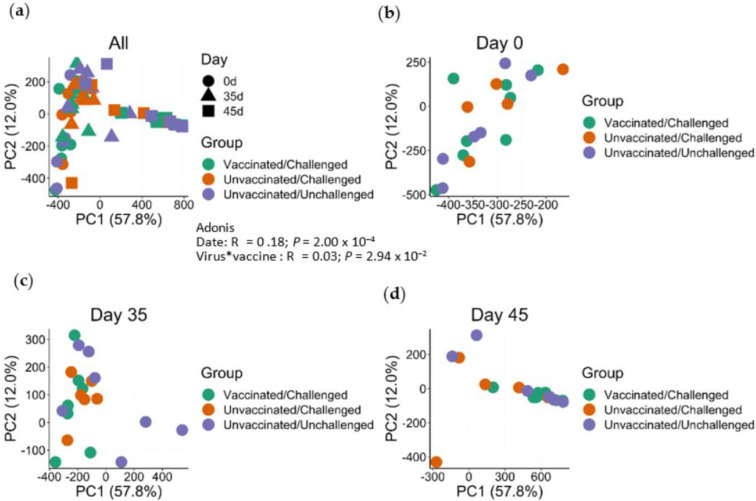
Fecal microbial beta diversity of Amplicon Sequence Variant (ASV) in different treatment groups of pigs. Principal component analysis (PCA, USA) plot using fecal samples from all days (**a**), 0 dpv (**b**), 35 dpv (**c**), and 45 dpv (**d**). The first two principal components (PCs) are plotted and colored according to the three design groups (Vaccinated/Challenged, Unvaccinated/Challenged, Unvaccinated/Unchallenged).

Next, we quantified how beta-diversity, a measurement of the similarity between two communities, varied across different treatment groups. Principal component analysis (PCA, USA) identified distinct shifts in the microbiome diversity across time (Figure 6a). Qualitatively, no group-specific clustering was apparent at 0 dpv (Figure 6b). By 35 dpv, vaccinated group 1 pigs visually appeared to begin separating from other groups of pigs (Figure 6c). After challenge, vaccinated and negative control pigs distinctly separated from group 2 unvaccinated/challenged pigs (Figure 6d). Supporting these qualitative findings, both time (R^2^ = 0.18; *p* = 2.00 × 10^−4^) and the interaction between the vaccination and virus challenge (R^2^ = 0.03; *p* = 2.94 × 10^−2^) significantly associated with microbiome diversity (Adonis). Together these results indicate that time, viral infection, and vaccination substantially impact microbiome diversity.

### 3.5. Evaluation of Associations between Microbial Genera Abundance, Vaccination, and Infection

**Figure 7:**
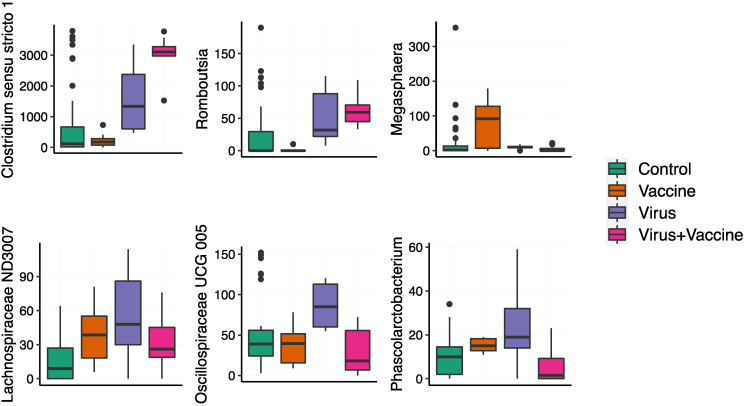
Fecal microbial genus level of taxonomic classification. Statistical analysis on genus level of taxonomic classification was conducted using a generalized linear mixed-effects model with a false discovery rate (FDR) controlled value below 0.2.

Negative binomial generalized linear mixed-effects models resolved the effects of vaccination and viral infection on the microbial genera abundance. A model selection procedure was used (glmmTMB) to compensate for temporal shifts in microbiota across the study. The result showed that PRRSV infection is associated with the increased abundance of Lachnospiraceae ND3007 group (z = 2.65, *p* = 7.99 × 10^−3^) and family Oscillospiraceae UCG-005 (z = 2.76, *p* = 5.73 × 10^−3^) (Figure 7). Other taxa were sensitive to interactions between the viral infection and vaccination, including Megasphaera (z = −2.46, *p* = 1.41 × 10^−2^), Romboutsia (z = 1.12, *p* = 7.30 × 10^−3^), Clostridium sensu stricto 1 (z = 3.29, *p* = 1.01 × 10^−3^), and Phascolarctobacterium (z = −3.49, *p* = 4.86 × 10^−4^). Overall, these observations suggest that vaccination alters the impacts of the PRRSV infection on the microbial genera abundances in the gut.

### 3.6. Microbial Correlations with Viremia and T Cell Immunity

To determine if the alterations in microbial community diversity are associated with responses to vaccination, we quantified monotonic relationships between viremia and immunological parameters using Spearman’s rank correlation. Viral load strongly correlated with both the primary axis of microbiome variation (PC1) value (ρ = −0.66, *p =* 1.60 × 10^−2^) and ASV richness (ρ = 0.63, *p* = 2.37 × 10^−2^). Richness was also correlated with PRRSV specific CD3^+^CD4^−^CD8^+^IFNγ^+^ T cell percentages (ρ = −0.65, *p =* 4.90 × 10^−2^). To determine if individual taxa associate with viremia or immune response, we next evaluated Spearman’s correlations between the genera abundance and viral load or the quantum of T cell subsets at 45 dpv. Five taxa, *Megasphaera* (ρ = 0.48), Lachnospiraceae ND3007 group (ρ = 0.59), *Prevotella* 9 (ρ = 0.49), *Ruminococcus* (ρ = 0.47), and *Monoglobus* (ρ = 0.55), all positively correlated with viral load (*p* < 0.05; Supplemental Table S1). Three microbial genera, Lachnospiraceae ND3007 group (ρ = 0.62, *p* =1.4 × 10^−2^), *Ruminococcus* (ρ = 0.59, *p* = 2.1 × 10^−2^), and *Monoglobus* (ρ = 0.68, *p* =5.0 × 10^−3^), positively correlate with activated IFNγ-secreting CD4^+^ T cells, and one genus, *Monoglobus* (ρ = 0.57, *p* = 3.0 × 10^−2^) positively associated with IFNγ-secreting CD8^+^ T cells. A single taxon, *Ligilactobacillus*, was negatively associated with both IFNγ-secreting CD4^+^ (ρ = −0.59, *p* = 2.1 × 10^−2^) and CD8^+^ (ρ = −0.60, *p* = 1.8 × 10^−2^) T cells. These results should be interpreted cautiously, however, as they failed to satisfy our false discovery control threshold of 0.2, indicating that many of these genera specific associations may be spurious. Regardless, together, these analyses suggest that specific microbiota may contribute to immune responses or be disrupted by infection or vaccination.

## 4. Discussion

Increasing studies have shown that gut microbiota modulates the immune response to vaccinations [11]. Vaccine efficacy even varies between individuals in human populations, which is largely due to distinct microbiota compositions [7]. The relationship between gut microbiota and vaccination is principally studied in mouse models or in human populations, but the studies of porcine gut microbiota impacts on vaccinations are still limited [25,26]. In our study, we performed 16S rRNA gene sequencing of fecal microbiome to explore potential associations of microbiota with PRRS vaccine efficacy. We found a high degree of temporal variance in microbiome diversity with early increases in diversity overcome by significantly decreased microbiome richness and Shannon diversity at later time points. One possible explanation for this observation is that successional patterns during porcine development are not linear [27]. Consistent with this hypothesis, previous studies have reported the developmental changes in the fecal microbiota for post-weaning piglets [28]. Interestingly, viral infection appeared to interfere with these natural patterns of microbiome succession during development, while vaccinated pig microbiomes developed a similar composition to that of negative control pigs. This suggests that viral infection may interrupt normal microbiome development. If this altered developmental trajectory persists, the impacts of early life viral infection may be propagated to later developmental time points or adulthood, similar to the impacts of antibiotic exposure [29]. Future work is warranted to clarify the longevity of viral impacts on microbiome composition and operation.

In our study, after the PRRSV infection of unvaccinated pigs, microbiome richness increased with marginal significance and microbial species diversity increased significantly. This finding is consistent with the previous report, in which PRRSV infection impacts the gut microbiome in a strain virulence-dependent fashion and the infection of a virulent Lena strain leads to higher species diversity as well as increased microbiome evenness and richness [30]. It was speculated that PRRSV infection impairs the microbiota composition and allows pathogenic bacteria to emerge and grow at low proportions [30]. However, another study also reported that increased gut microbiota diversity leads to improved growth outcomes and better confrontation against disease infections [17,31]. The controversial conclusions may be due to an experimental design of PRRSV/PCV2 co-infection and a lack of negative control pigs [30]. Additionally, different pig breeds and facilities used may result in microbiome composition and diversity differences [32].

In our study, gut microbiome diversity (alpha and beta) in vaccinated pigs was qualitatively similar to that of naïve pigs. As opposed to increased diversity in infected pigs, the decreased microbiome diversity in vaccinated pigs is similar to naïve controls and this reduced diversity correlates with reduced viral shedding. Reduced gut microbiome diversity was also reported to be associated with enhanced humoral immunity in pigs injected with cholera toxin subunit B (CTB) and tetanus toxoid [33]. However, increased diversity was previously reported to be associated with high growth outcomes in PRRS vaccinated pigs that co-challenged with PRRSV and PCV2b [34]. These disparate results may be due to specific taxonomic changes that underlie the association between altered diversity and host immune responses. In our study, we identified several genera that are associated with viral loads and T cell responses. The high false discovery rate linked with these observations means that these associations should be interpreted cautiously; however, similar observations have been made in previous studies [31], suggesting that at least some of the correlations we observed here are likely valid. Thus, it is possible that disruption of the gut microbiome may affect immune responses to PRRS vaccination. Further studies are needed to identify the specific microbial genera that affect the pathophysiology of the disease and the efficacy of vaccination.

## 5. Conclusions

In conclusion, the PRRSV killed vaccine prepared in this study provided partial protection against PRRSV challenge in a nursery pig model. The protective effect of the vaccine results in restored microbiome diversity in the gut of the pigs. The microbial signatures correlating with vaccine efficacy and immunogenicity were explored. This study provides additional evidence on the association of changes in the gut microbiome with PRRSV vaccination and infection. It paves the road for employing microbiota in the development of more efficient PRRS vaccines.

## Data Availability

Data sharing is not applicable to this article.

## Supplementary Materials

The following supporting information can be downloaded at: https://www.mdpi.com/article/10.3390/v14051081/s1, Table S1. Microbial taxa correlations with T cell immunity and viremia.
