# Supplementary material for: Effect of Killed PRRSV Vaccine on Gut Microbiota Diversity in Pigs
Source: Viruses. 2022 May 18;14(5):1081. doi: 10.3390/v14051081 (PMC9145812; doi:10.3390/v14051081)
Supplement: Supplementary file 1 [file viruses-14-01081-s001.zip › viruses-1685853-supplementary.pdf]

Table S1. Microbial taxa correlations with T cell immunity and viremia

Taxa	Host factor	p-value[a]	ρ[b]
Ligilactobacillus	CD8	0.018	-0.600
Monoglobus	CD8	0.026	0.572
Lachnospiraceae.ND3007.group	CD4	0.014	0.617
Ruminococcus	CD4	0.021	0.588
Ligilactobacillus	CD4	0.021	-0.590
Monoglobus	CD4	0.005	0.683
Megasphaera	viremia	0.046	0.476
Lachnospiraceae.ND3007.group	viremia	0.008	0.600
Prevotella_9	viremia	0.039	0.490
Ruminococcus	viremia	0.047	0.474
Monoglobus	viremia	0.019	0.546

[a] p-value less than 0.05 considered as statistic significance.
[b] Spearman's correlation coefficient between microbial taxa and host factor, negative number indicates negative correlation, while positive number indicates positive correlation.
